# Educational Attainment Influences Levels of Homozygosity through Migration and Assortative Mating

**DOI:** 10.1371/journal.pone.0118935

**Published:** 2015-03-03

**Authors:** Abdel Abdellaoui, Jouke-Jan Hottenga, Gonneke Willemsen, Meike Bartels, Toos van Beijsterveldt, Erik A. Ehli, Gareth E. Davies, Andrew Brooks, Patrick F. Sullivan, Brenda W. J. H. Penninx, Eco J. de Geus, Dorret I. Boomsma

**Affiliations:** 1 Department of Biological Psychology, VU University Amsterdam, Amsterdam, The Netherlands; 2 Neuroscience Campus Amsterdam, Amsterdam, The Netherlands; 3 EMGO+ Institute for Health and Care Research, Amsterdam, The Netherlands; 4 Avera Institute for Human Genetics, Avera McKennan Hospital & University Health Center, Sioux Falls, South Dakota, United States of America; 5 Department of Genetics, Rutgers, The State University of New Jersey, Piscataway, New Jersey, United States of America; 6 Department of Genetics, University of North Carolina, Chapel Hill, North Carolina, United States of America; 7 Department of Psychiatry, VU University Medical Center, Amsterdam, Netherlands; Johns Hopkins Bloomberg School of Public Health, UNITED STATES

## Abstract

Individuals with a higher education are more likely to migrate, increasing the chance of meeting a spouse with a different ancestral background. In this context, the presence of strong educational assortment can result in greater ancestry differences within more educated spouse pairs, while less educated individuals are more likely to mate with someone with whom they share more ancestry. We examined the association between educational attainment and *F*_roh_ (= the proportion of the genome consisting of runs of homozygosity [ROHs]) in ~2,000 subjects of Dutch ancestry. The subjects’ own educational attainment showed a nominally significant negative association with *F*_roh_ (*p* = .045), while the contribution of parental education to offspring *F*_roh_ was highly significant (father: *p* < 10^-5^; mother: *p* = 9×10^-5^), with more educated parents having offspring with fewer ROHs. This association was significantly and fully mediated by the physical distance between parental birthplaces (paternal education: *p*_*mediation*_ = 2.4 × 10^-4^; maternal education: *p*_*mediation*_ = 2.3 × 10^-4^), which itself was also significantly associated with *F*_roh_ (*p* = 9 × 10^-5^). Ancestry-informative principal components from the offspring showed a significantly decreasing association with geography as parental education increased, consistent with the significantly higher migration rates among more educated parents. Parental education also showed a high spouse correlation (Spearman’s ρ = .66, *p* = 3 × 10^-262^). We show that less educated parents are less likely to mate with the more mobile parents with a higher education, creating systematic differences in homozygosity due to ancestry differences not directly captured by ancestry-informative principal components (PCs). Understanding how behaviors influence the genomic structure of a population is highly valuable for studies on the genetic etiology of behavioral, cognitive, and social traits.

## Introduction

Non-random mating can create systematic differences in parental relatedness, which can have a direct and detectable impact on genome-wide homozygosity in subsequent generations. Non-random mating in human populations can be driven by heritable behavioral traits. It is important to understand how behavior has influenced our genetic variation in order to successfully conduct and interpret studies that aim to understand the reverse, namely how genetic variation influences behavior. In the Netherlands for example, the consequences of continuous religious assortment during the last ~400 years and the relatively recent secularization are detectable through homozygosity differences between religious and non-religious groups. Such systematic differences can cause spurious associations between homozygosity and traits related to religiosity [1].

Educational attainment (EA) is another complex trait that may induce systematic differences in parental relatedness. Education shows considerable levels of assortment [2,3,4,5,6]. In addition, individuals with a higher education are more likely to have moved away from their birthplace, making the physical distance between them and their family members two to three times greater than for individuals with a lower education [7]. When ancestry shows high correlations with geography, like in the Netherlands [8,9], these behaviors may increase the chance for higher educated individuals to mate with someone with a different ancestral background, making their offspring more outbred, while less educated spouse pairs are more likely to share more ancestry.

EA and its etiology have been widely studied. EA is heritable in populations in which it has been studied, with estimates ranging from ~20% to ~80% and increasing over time [10,11]. EA is associated with many other traits, such as psychiatric disorders [12,13], personality [14], life expectancy [15], overall health [16], and is especially deeply related to IQ [17,18]. IQ is predictive for EA, and is a heritable complex trait [19,20] of which the underlying genetic etiology is largely unknown. This makes EA itself an appealing trait for genetic association studies since it is more feasible to measure on a large scale than IQ [21,22]. Higher cognitive function has recently been associated with increased homozygosity levels in a representative UK sample [23], which is in the opposite direction of what one would expect assuming that individuals with a higher education are more likely to mate with someone with different ancestry. Assortative mating on cognitive function was posed as a potential explanation for this finding, where assortment among individuals with higher cognitive ability may have induced increased homozygosity for loci that contribute to higher cognitive ability.

The current study examines how migration, ancestral background, and the proportion of the offspring genome consisting of runs of homozygosity (ROHs: multiple contiguous homozygous single nucleotide polymorphisms [SNPs]), vary systematically between different levels of own and parental EA. The proportion of the genome consisting of ROHs is quantified by *F*_roh_, which has demonstrated to be a powerful measure for shared ancestry of genetic haplotypes, and is generally used to study the deleterious effects of inbreeding in humans and other animals [24]. If parents with a higher education have higher migration rates and tend to select mates with different ancestral backgrounds through assortative mating, we expect their offspring to show lower *F*_roh_ levels as well as weaker associations between ancestry-informative PCs and geography.

Data from a population cohort of ~2,000 unrelated subjects of Dutch ancestry included the EA of the participants and their parents, genome-wide SNPs, ancestry informative principal components (PCs), current living address, birthplace, parental birthplace, and religious affiliation.

## Materials and Methods

### Participants

Genotyped subjects were registered at the Netherlands Twin Register (NTR [25], N = 6,685; 2,678 males and 4,007 females). The NTR subjects were randomly sampled from twin families across the Netherlands. Analyses were done on unrelated individuals only. Unrelated individuals were chosen using GCTA [26], by excluding one of each pair of individuals with an estimated genetic relationship of >0.025 (i.e., more related than third or fourth cousin). Only individuals with Dutch ancestry were included. Individuals with a non-Dutch ancestry were identified by projecting PCs from 1000 Genomes populations on the dataset, and with additional help of the birth country of the parents. This procedure is described in more detail elsewhere [8].

This study was approved by the Central Ethics Committee on Research Involving Human Subjects of the VU University Medical Centre, Amsterdam, an Institutional Review Board certified by the US Office of Human Research Protections (IRB number IRB-2991 under Federal-wide Assurance-3703; IRB/institute codes, NTR 03–180). All subjects provided written informed consent.

### Phenotypes

EA was measured longitudinally with the question “*What is the highest educational level that you have finished*?”, “*What is the highest educational level that your father has finished*?”, and “*What is the highest educational level that your mother has finished*?”. The answer categories varied per survey, but could all be recoded into the following four categories: 1) Primary Education; 2) Secondary Education (VMBO, LBO, MAVO, lower secondary); 3) Higher Secondary Education (MBO, HAVO/VWO, higher secondary); 4) Tertiary Education (HBO, university, PhD). EA was available for 2,089 unrelated genotyped Dutch subjects, paternal EA was measured for 2,067 unrelated genotyped Dutch subjects, and maternal EA was available for 2,075 unrelated Dutch subjects (largely in the same subjects: 2,026 individuals had their own, their paternal, and maternal EA available).

Information on birthplace was available from survey and from city council register data for 1,892 unrelated genotyped Dutch subjects, paternal birthplace for 1,465 subjects, and maternal birthplace for 1,618 unrelated Dutch subjects; 1,371 individuals had both their own and paternal birthplace available, 1,513 individuals had their own and maternal birthplace available, 1,312 had both parental birthplaces available, and 1,227 individuals had their own, their paternal, and maternal birthplace available. Distance between birthplaces was computed with a purpose written perl script using the algorithm available on http://www.geodatasource.com/developers/perl. Data on parental birthplace were extracted from city council registers and were available for parents that were alive after 1994. Parental birthplace distance and distance between own and parental birthplace were analyzed in the sections “*Migration distance and EA*” and “*Migration distance and F*_*roh*_”, i.e., the analyses from these two sections have mainly been run on more recent generations that are on average more highly educated. The effects of parental migration however can also be deducted from the analyses in the section “*Association between geography and ancestry per parental educational level*“, where parental EA in combination with *own* birthplace and ancestry-informative PCs were analyzed; the results from this section were similar after splitting up the sample into subjects with and subjects without data on parental birthplace.

The assessment of religion and city size are described in detail elsewhere [1]. Sample sizes of individuals that had EA, religion, and city size available (i.e., were included in the statistical analyses) are given in the Results section and the Tables for each analysis.

### Genotyping, QC, and ancestry-informative PCs

Genotyping was performed on the Affymetrix Human Genome-Wide SNP 6.0 Array according to the manufacturer’s protocol. Methods for blood and buccal cell collection, genomic DNA extraction, genotyping, and QC have been described previously [1,27,28]. Only autosomal SNPs were analyzed. After QC, 498,592 SNPs remained.

Ancestry-informative PCs were computed with EIGENSTRAT [29] on 5,166 unrelated subjects with Dutch ancestry, which also included subjects from the Netherlands Study of Depression and Anxiety (NESDA) [30]. The ancestry-informative PCs and their computation are described in detail elsewhere [8].

### ROHs and F_roh_

ROHs were called using Plink [31]. A recent study comparing several software packages designed for this goal concluded that Plink predicts autozygous stretches optimally [32], using simulated data based on the Affymetrix 6.0 chip, making their density of SNPs in linkage disequilibrium (LD) close to ours We followed the recommendations from this study in calling ROHs: (1) SNPs were pruned for LD (window size = 50, number of SNPs to shift after each step = 5, based on a variance inflation factor [VIF] of 2), resulting in 131,325 SNPs; (2) an ROH was defined as ≥65 consecutive homozygous SNPs with no heterozygote calls allowed. *F*_roh_ is an overall measure of the proportion of the autosome in ROHs, which is calculated as the total length of ROHs summed for each individual, and then divided by the total SNP-mappable autosomal distance (2.77 × 10^9^ bases).

### Statistical analyses

#### Migration distance and EA

The relation between birthplace distances and EA was investigated firstly with a one-way ANOVA in IBM SPSS Statistics 20, with birthplace distance as the dependent variable and EA as the independent variable. Four tests were performed: 1) distance between own and paternal birthplace as the dependent variable and paternal EA as independent variable; 2) distance between own and maternal birthplace as the dependent variable and maternal EA as independent variable; 3) distance between parental birthplaces as the dependent variable and paternal EA as independent variable; 4) distance between parental birthplaces as the dependent variable and maternal EA as independent variable. Post-hoc tests were then conducted with t-tests comparing birthplace distances between each two consecutive educational levels, computed in IBM SPSS Statistics 20.

#### Educational assortment and parent-offspring correlations

The chi-squared test and Spearman’s ρ were computed in IBM SPSS Statistics 20 to test for the assortment on EA.

#### EA and F_roh_

The R^2^ change (= difference in explained variation of *F*_roh_) was computed between multiple regressions on *F*_roh_ with and without EA as a predictor (i.e., own or parental EA). The regressions included as predictors: the three PCs reflecting ancestry (correlated significantly with geography: PC1 = North-South PC, PC2 = East-West PC, PC3 = middle-band PC) [8], city size (dichotomous, i.e., living in a city with population size >100k), and religion. To evaluate the presence of a birth cohort effect, the analyses were repeated including year of birth as an additional predictor. To correct for the non-normal distribution of *F*_roh,_ *F*_roh_ was permuted 100,000 times (i.e., *F*_roh_ was randomly shuffled across subjects 100,000 times and the R^2^ change was re-computed in all these random “null” datasets, after which an empirical *p*-value was computed for the R^2^ change by dividing the rank of the *p*-value of the real dataset among the “null” datasets by 100,000; all reported empirical *p*-values were almost identical to the *p*-value of the real dataset). These analyses were done in a purpose written perl script, using the PDL::Stats::GLM perl module (see http://search.cpan.org/~maggiexyz/PDL-Stats-0.6.2/GLM/glm.pp).

#### Migration distance and F_roh_

**Fig 1:**
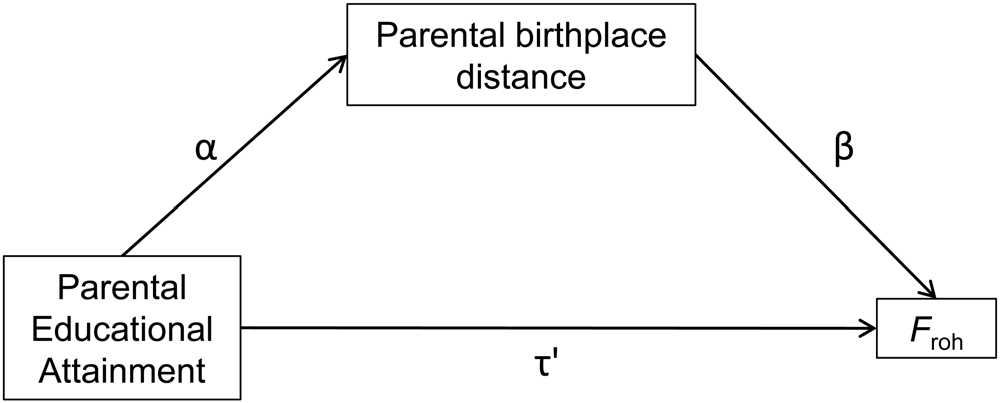
Representation of the mediation model described in the section “*Migration distance and F*_roh_”. The five covariates included in the model (the three PCs reflecting ancestry, city size, and religion) are not shown in this Figure.

The R^2^ change (= difference in explained variation of *F*_roh_) was computed between multiple regressions on *F*_roh_ with and without the distance between birthplaces as a predictor. The regressions included as predictors: the three PCs reflecting ancestry, city size, religion, and EA. *F*_roh_ was permuted 100,000 times. These analyses were done in a purpose written perl script, using the PDL::Stats::GLM perl module. In addition, a Sobel test was conducted in LISREL, where the significance of the mediation effect of parental birthplace distance on the association between parental EA and *F*_roh_ was tested. The null hypothesis was αβ = 0, where α represents the relationship between parental EA and parental birthplace distance, and β represents the relationship between birthplace distance and *F*_roh_ (see Fig. 1); τ’ (which represents the relationship between parental EA and *F*_roh_) was freely estimated in this model. We also conducted a full mediation test, where we tested whether τ’ = 0 when including α and β in the model, i.e., whether the association between parental EA and *F*_roh_ remained significant after including parental birthplace distance as a mediator. Both the Sobel test and the full mediation test included as covariates: the three PCs reflecting ancestry, city size, and religion.

#### Association between geography and ancestry per parental educational level

The influence of parental educational level on correlations between ancestry-informative PCs of the offspring and geographic location was tested using full information maximum likelihood estimation in OpenMx [33], separately for maternal and paternal educational levels and the two ancestry-informative PCs. We approximated the effect of parental EA on the correlations between PC-values and geographic location with the following linear model: ρ = ρ_0_ + education*ρ_1_, where ρ is the correlation between PC and latitude/longitude. The null hypothesis ρ_1_ = 0 was tested by mean of the likelihood ratio test.

## Results

### Migration distance and EA

**Fig 2:**
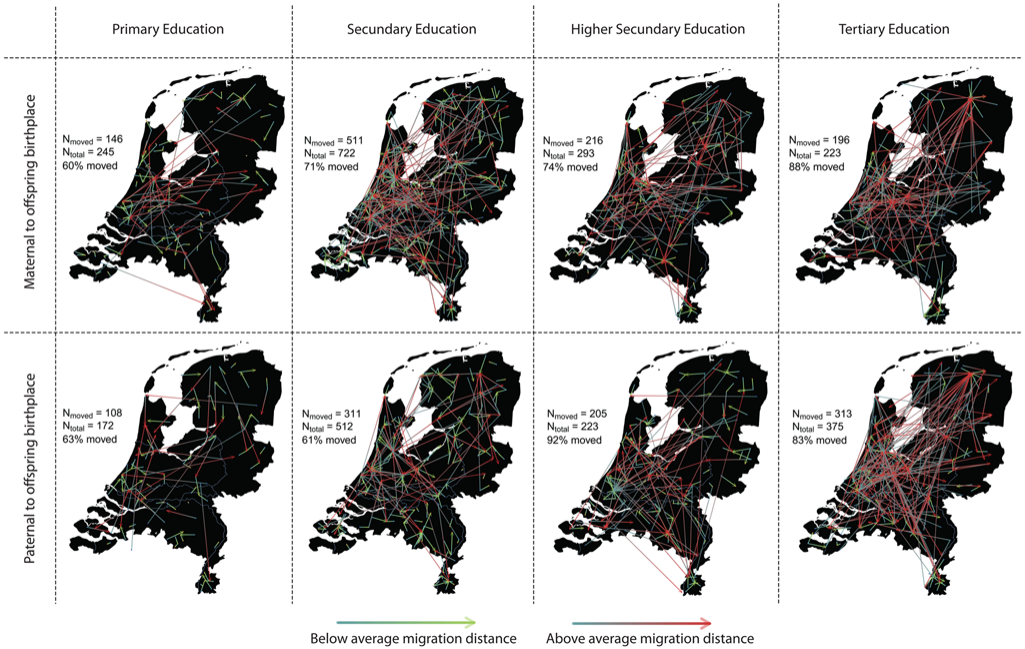
Migrations from the parental birthplace to the offspring birthplace. The average distance the colors are based on are: father: 28.47 km (SD = 44.45); mother: 30.16 km (SD = 44.45). The difference between the moving distance of fathers with a Secondary Education and fathers with a Tertiary Education is best suited to visualize the effect because of the almost equal sample sizes with respect to individuals plotted (i.e., moved) and the significant increase of moving distance (see Table 1); also note that fathers with Secondary Education have >25% measurements in total, which is another indicator of the difference in migration levels.

The distance between parental birthplace and own birthplace was significantly associated with EA (distance between own and paternal birthplace: *p* = 8.9 × 10^-30^, N = 1,349; distance between own and maternal birthplace: *p* = 1.2 × 10^-26^, N = 1,483). Post-hoc tests showed that this association is mainly driven by a significantly increasing migration distance as the educational level exceeds the Secondary Education (see Table 1 and Fig. 2), with parents with a Tertiary Educational level having moved more than twice the distance than parents with Primary or Secondary Educational levels. The same effect was observed for the distance between paternal and maternal birthplace for both paternal (*p* = 2.8 × 10^-13^; N = 1,294) and maternal (*p* = 1.7 × 10^-16^; N = 1,291) educational levels (see Table 1), showing that more highly educated individuals are more likely to mate with a partner from a different geographic region.

**Table 1:** Mean distance in km between birthplaces, and *p*-values of t-tests testing the difference in birthplace distance between parental educational attainment (EA) levels.

EA level	Mean distance (km)	*p*-value difference test
*Mean distance between paternal and own birthplace (km) per paternal EA level*:		
1. Primary	19.2 (*SD* = *32*.*4*;*N* = *172*)	-
2. Secondary	16.3 (*SD* = *30*.*8*;*N* = *512*)	.29 (vs. 1)
3. Higher secondary	28.7 (*SD* = *46*.*0*;*N* = *291*)	4.5×10^-5^ (vs. 2)
4. Tertiary	49.8 (*SD* = *55*.*3*;*N* = *375*)	1.1×10^-7^ (vs. 3)
*Mean distance between maternal and own birthplace (km) per maternal EA level*:		
1. Primary	19.2 (*SD* = *34*.*7*;*N* = *245*)	-
2. Secondary	24.1 (*SD* = *37*.*8*;*N* = *722*)	. 07 (vs. 1)
3. Higher secondary	34.6 (*SD* = *44*.*2*;*N* = *293*)	3.6×10^-4^ (vs. 2)
4. Tertiary	56.7 (*SD* = *52*.*6*;*N* = *223*)	6.9×10^-7^ (vs. 3)
*Mean distance between paternal and maternal birthplace (km) per paternal EA level*:		
1. Primary	22.8 (*SD* = *34*.*9*;*N* = *144*)	-
2. Secondary	23.5 (*SD* = *39*.*7*;*N* = *483*)	.85 (vs. 1)
3. Higher secondary	34.0 (*SD* = *49*.*8*;*N* = *284*)	2.7×10^-3^ (vs. 2)
4. Tertiary	46.4 (*SD* = *50*.*5*;*N* = *383*)	1.6×10^-3^ (vs. 3)
*Mean distance between paternal and maternal birthplace (km) per maternal EA level*:		
1. Primary	25.5 (*SD* = *47*.*8*;*N* = *145*)	-
2. Secondary	24.8 (*SD* = *39*.*9*;*N* = *641*)	. 86 (vs. 1)
3. Higher secondary	36.2 (*SD* = *46*.*6*;*N* = *283*)	4×10^-4^ (vs. 2)
4. Tertiary	54.7 (*SD* = *52*.*7*;*N* = *222*)	4.7×10^-5^ (vs. 3)

### Educational assortment and parent-offspring correlations

Parental educational levels showed a high spouse correlation (Spearman’s ρ = .66, *p* = 3 × 10^-262^, N = 2,058; see Table 2). The majority of the parents (58.5%) shared the same educational level. The only other spouse pair combinations showing higher observed frequencies than expected are fathers with a Higher Secondary Education and mothers with a Secondary Education, or fathers with a Tertiary Education and mothers with a Higher Secondary Education (Table 2), a gender-asymmetrical pattern known as hypergamy [34]. The correlation between parental EA and offspring EA was considerably lower than the spouse correlation, but still highly significant (paternal EA and offspring EA: Spearman’s ρ = .40, *p* = 1.4 × 10^-78^, N = 2,035; maternal EA and offspring EA: Spearman’s ρ = .38, *p* = 3.6 × 10^-71^, N = 2,041).

**Table 2:** Crosstab of 2,058 spouse pairs and their educational attainment, including χ^2^ test and Spearman’s rank correlation coefficient.

χ^2^ (9) = 1496.89, *p* <. 001, Spearman’s ρ = .664		*Mother*			
		Primary education	Secondary education	Higher secondary education	Tertiary education
*Father*	Primary education	**273 (78.2)**	79 (165.3)	11 (70.8)	8 (56.6)
	Secondary education	110 (154.4)	**528 (326.2)**	70 (139.8)	24 (111.7)
	Higher secondary education	38 (86.5)	**190 (182.7)**	**151 (78.3)**	31 (62.6)
	Tertiary education	13 (114.9)	120 (242.8)	**161 (104.1)**	**251 (83.2)**

The numbers between brackets is the expected number of spouse pairs in that cell under the null hypothesis of no assortment. Observed values higher than the expected values are in bold.

### EA and F_roh_

The subjects’ own EA showed a nominally significant negative association with *F*_roh_ (*p* = .045, N = 2,007). The association between offspring *F*_roh_ and parental EA was highly significant (father: *p* < 10^-5^, N = 1,989; mother: *p* = 9×10^-5^, N = 1,995), with more highly educated parents having offspring with lower *F*_roh_ levels (see Tables 3 and 4). Multiple confounders were accounted for in all regressions: we included ancestry-informative PCs, city size (i.e., living in a city with population size >100k), and religion (see reference [1] for more details on the relationship between these variables and *F*_roh_). Religion significantly contributed to *F*_roh_ variation after including parental EA to the regression, and this significance diminished only slightly after the inclusion of EA in the regression, indicating a (partly) independent effect of religion and EA on *F*_roh_ (see Table 4).

**Table 3:** Mean *F*_*roh*_ of the offspring, standard deviation, and sample sizes for each educational attainment (EA) group.

EA level	Offspring EA	Paternal EA	Maternal EA
Primary	.00192 (*SD* = .*003*;*N* = *74*)	.00200 (*SD* = .*003*;*N* = *372*)	.00184 (*SD* = .*003*;*N* = *439*)
Secondary	.00180 (*SD* = .*003*;*N* = *368*)	.00177 (*SD* = .*004*;*N* = *734*)	.00177 (*SD* = .*004*;*N* = *925*)
Higher secondary	.00170 (*SD* = .*003*;*N* = *659*)	.00149 (*SD* = .*003*;*N* = *413*)	.00127 (*SD* = .*002*;*N* = *397*)
Tertiary	.00141 (*SD* = *003*;*N* = *988*)	.00108 (*SD* = *001*;*N* = *548*)	.00100 (*SD* = *001*;*N* = *314*)

**Table 4:** Standardized betas (and *p*-values between brackets) in the bottom six rows for each of the predictors included in the linear regressions with offspring *F*_roh_ as a dependent variable, as well as the R^2^ change (= increase in explained variance after adding educational attainment (EA) as a predictor) and its empirical *p*-value from 100k permutations in the top row.

Predictors regressed on offspring *F*_roh_	*Regression excluding and including offspring EA as a predictor (N = 2*,*007*): R^2^ change after including offspring EA = .002 (empirical *p* = .045)		*Regression excluding and including paternal EA as a predictor (N = 1*,*989*): R^2^ change after including paternal EA = .009 (empirical *p* < 10^-5^)		*Regression excluding and including maternal EA as a predictor (N = 1*,*995*): R^2^ change after including maternal EA = .008 (empirical *p* = 9×10^-5^)	
	*Excluding offspring EA*	*Including offspring EA*	*Excluding paternal EA*	*Including paternal EA*	*Excluding maternal EA*	*Including maternal EA*
EA	NA	-.0411 (.046)	NA	-.0884 (2.0×10^-5^)	NA	-.0803 (8.8×10^-5^)
PC1 (North-South)	.0744 (1.8×10^-4^)	.0735 (2.2×10^-4^)	.0759 (1.4×10^-4^)	.0716 (3.3×10^-4^)	.0675 (6.3×10^-4^)	.0658 (8.4×10^-4^)
PC2 (East-West)	.0531 (6.1×10^-3^)	.0530 (6.2×10^-3^)	.0553 (4.4×10^-3^)	.0577 (2.9×10^-3^)	.0593 (2.3×10^-3^)	.0637 (8.9×10^-4^)
PC3 (Middle-Band)	.0232 (.252)	.0224 (.269)	.0180 (.378)	.0168 (.407)	.0256 (.203)	.0239 (.233)
Religion (yes/no)	.1252 (4.0×10^-3^)	.1228 (4.7×10^-3^)	.1239 (4.4×10^-3^)	.1098 (.011)	.1214 (4.6×10^-3^)	.1030 (.016)
City Variable	-.0324 (.141)	-.0266 (.230)	-.0314 (.152)	-.0167 (.449)	-.0312 (.150)	-.0185 (.397)

To evaluate whether the age difference between the genotyped subjects and their parents contributed to the difference between the effects of the subjects’ own education and the parental education, the analyses were repeated only including individuals that were at an age where they were more likely to have completed their education. The analyses were run once only including subjects with age > 25, and once including only ages > 30. Both these analyses gave a non-significant result for the subjects’ own EA (age>25: *p* = .065, N = 1,641; age>30: *p* = .075, N = 1,401), while parental EA remained significant (*father*: age>25: *p* = 9.9 × 10^-4^, N = 1,610; age>30: *p* = 1.6 × 10^-3^, N = 1,371; *mother*: age>25: *p* = 3.8 × 10^-3^, N = 1,616; age>30: *p* = .046, N = 1,376). Accounting for year of birth in order to evaluate the presence of a cohort effect also still results in a non-significant association between own EA and *F*_roh_ with own year of birth added as a predictor (*p* = .181, N = 1,984), and a significant association between parental EA and *F*_roh_ with the parental year of birth added as an additional predictor (father: *p* = 3.5 × 10^-3^, N = 1,401; mother: *p* = 7.2 × 10^-3^, N = 1,534).

### Migration distance and F_roh_

A larger distance between paternal and maternal birthplace was significantly associated with lower *F*_roh_ (*p* = 9 × 10^-5^, N = 1,263). A larger distance between own birthplace and parental birthplace also resulted in significantly lower *F*_roh_ levels (paternal and own birthplace distance: *p* = 5.3 × 10^-3^, N = 1,317; maternal and own birthplace distance: *p* = 9.5 × 10^-3^, N = 1,445). After including the distance between the paternal and maternal birthplaces as a predictor, parental EA was no longer significantly associated with *F*_roh_ (paternal EA: *p* = .077, N = 1,246; maternal EA: *p* = .134, N = 1,242), while the birthplace distance still contributed significantly to *F*_roh_ variation (in regression including paternal EA: *p* = 4.6 × 10^-5^, in regression including maternal EA: *p* = 3.1 × 10^-5^).

To test the significance of the mediation effect, we performed a Sobel test (Fig. 1), including the same covariates as the regression analyses. For both parents, parental birthplace distance significantly mediated the association between parental EA and *F*_roh_ (paternal EA: *p* = 2.4 × 10^-4^; maternal EA: *p* = 2.3 × 10^-4^). In addition, we conducted a full mediation test, i.e., we tested whether there is still an association left between parental EA and *F*_roh_ after mediation by parental birthplace distance. After including parental birthplace distance as a mediator in the model, parental EA was no longer associated with *F*_roh_ (paternal EA: *p* = .06; maternal EA: *p* = .12), indicating a full mediation effect of parental birthplace distance. These results show that the association between *F*_roh_ and parental EA is explained by parents with a higher education tending to have more different ancestries than less educated parents because of higher migration levels.

### Association between geography and ancestry per parental educational level

**Fig 3:**
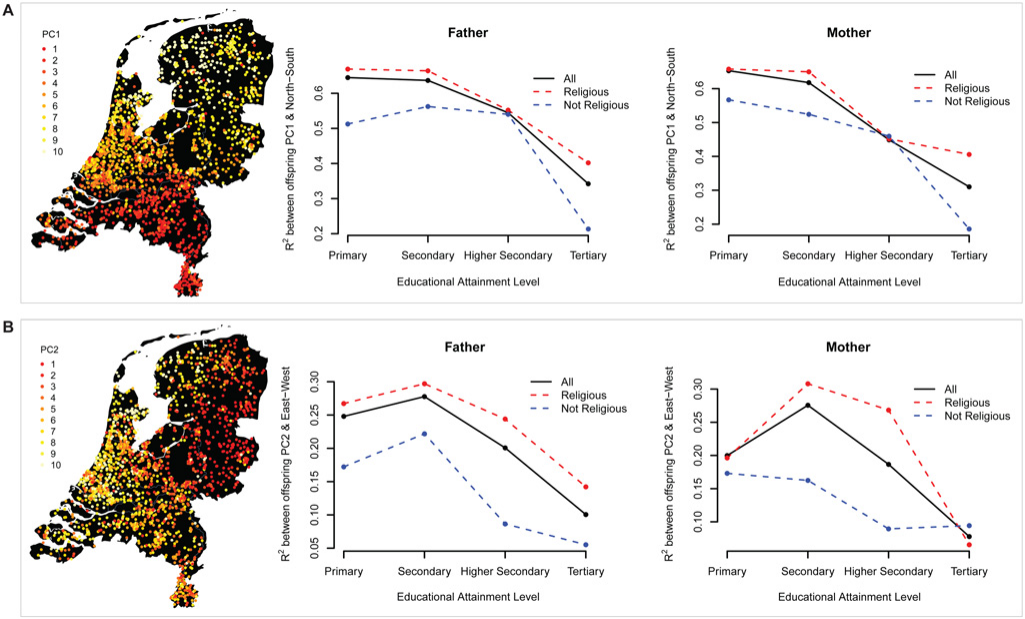
Association between geography and ancestry per parental educational attainment level. *A*—Left: geographic distribution of PC1 (N = ~5,000 unrelated Dutch subjects), where the mean PC1 value per postal code (current living address) was computed, divided into 10 percentiles, and plotted. Right: two plots showing the explained variance (R^2^) of the offspring’s PC1 by the North-South gradient based on the offspring’s birthplace, per parental educational group. *B*—Left: geographic distribution of PC2. Right: two plots showing R^2^ between offspring PC2 and the East-West gradient based on offspring’s birth place.

PCs from genome-wide single-nucleotide polymorphisms (SNPs) capture ancestral background, and show high correlations with geography within the Netherlands and other countries [8,35,36,37]. In the current dataset, the first PC correlates. 74 with the North-South gradient based on birthplace, and the second PC correlates. 46 with the East-West gradient (N = 1,892). The correlations between PCs and geographic location significantly differed between educational groups (PC1 for paternal EA: *p* = 2.2 × 10^-12^; PC1 for maternal EA: *p* = 8.2 × 10^-12^; PC2 for paternal EA: *p* = 2.5 × 10^-4^; PC2 for maternal EA: *p* = 3.5 × 10^-4^). Fig. 3 shows a decreasing association between the PCs and geography as the parental education increases. We approximated this decrease with a linear trend (ρ = ρ_0_ + EA*ρ_1_, where ρ is the correlation between PC and latitude/longitude, and EA is coded by 0, 1, 2, and 3), which gave us significant negative parameter estimates for ρ_1_: PC1 for paternal EA: ρ_1_ = -.06, *p* = 1.7 × 10^-11^; PC1 for maternal EA: ρ_1_ = -.07, *p* = 8.9 × 10^-12^; PC2 for paternal EA: ρ_1_ = -.06, *p* = 1.7 × 10^-4^; PC2 for maternal EA: ρ_1_ = -.05, *p* = 6.5 × 10^-3^. This would be expected if parents of subjects with higher EA tended to either live in a different geographic area than their ancestors, or that their partners live in a different geographic area than their ancestors. The effect is still visible after splitting up the sample into a religious and non-religious group (Fig. 3). The non-religious group shows an overall weaker association between geography and the PCs, consistent with previously observed lower *F*_roh_ levels in the non-religious group in the Netherlands [1], and suggesting migration may have also played a role in the homozygosity differences between religious and secular groups.

## Discussion

The proportion of the autosomal genome in ROHs (*F*_roh_) shows a nominally significant negative association with EA. In the absence of data on parental EA, geographic mobility, and ancestry, this observation could have been interpreted as the result of deleterious effects of inbreeding on cognitive ability, which would fit the existing hypotheses [38,39,40,41]. The effect was considerably more significant however when associating *F*_roh_ with paternal or maternal EA. We investigated whether this could be explained by a combination of migration and educational assortment. Ancestry correlates highly with geography in the Netherlands due to relatively low levels of within-country migrations in recent history [8]. Individuals with higher EA were significantly more likely to have migrated away from their birthplace and to mate with a partner from a different geographic region. In this context, educational assortment increases the chance for more highly educated individuals to mate with genetically more dissimilar partners, lowering the number of homozygous alleles transmitted to their offspring, while less educated individuals would have been more likely to mate closer to their ancestry. The association between *F*_roh_ and parental EA disappears after correcting for the distance between the paternal and maternal birthplaces, which itself was also significantly associated with *F*_roh_. Additional mediation tests showed that the association between *F*_roh_ and parental EA was indeed fully mediated by the parental birthplace distance. This is in line with the declining correlation between ancestry-informative PCs and the geographic location of the birthplace in subjects with more highly educated parents (Fig. 3). The same trend is visible after splitting up the sample in a religious and non-religious group, with the non-religious group showing consistently lower correlations between PCs and geography, suggesting migration may have also played a role in the *F*_roh_ differences between the religious and secular groups previously observed in this sample [1].

It has been suggested that outbreeding may explain the large increase in IQ from one generation to the next since the start of the 20^th^ century in many parts of the world (also known as the “Flynn effect”) [42]. If outbreeding is indeed associated with increased cognitive abilities, which would likely require much larger sample sizes to detect [24], the increased heterozygosity in subsequent generations caused by the non-random migration and mating patterns described in this study may have led (or could lead) to a feedback loop, which may contribute to an increase in IQ in each following generation.

A study in a UK sample found a nominally significant association between cognitive ability, which is predictive for EA, and *F*_roh_ in the opposite direction, with increased *F*_roh_ levels in individuals with higher cognitive ability [23]. Considering the high correlation between IQ and EA [17,18], the significant association between genetic variation and geography in the UK [43,44], and higher migration rates for more highly educated individuals within the UK [45,46], we would have expected an association between cognitive ability and *F*_roh_ in the same direction as EA shows in the Dutch population. The authors hypothesized that the ROHs causing this association harbor causal variants that have become homozygous through assortative mating on cognitive function. This difference in results is reminiscent of the difference in direction between populations for the association between *F*_roh_ and major depressive disorder (MDD) for which the UK and the Netherlands also showed an opposite direction of effect [1,47]. The MDD-*F*_roh_ association disappeared in the Dutch sample after correcting for systematic differences in parental relatedness between religious and non-religious groups. These phenomena illustrate the importance of the impact of complex social, demographic, and historical processes on the genomic structure of populations. The fact that the offspring education was much less significantly associated with offspring *F*_roh_ than the parents’ education and that the association disappeared after correcting for the distance between parental birthplaces strongly suggests that the effects we observed in the Dutch population do not reflect systematic differences in the frequency of causal genetic variants. Further analyses in a more deeply phenotyped and representative UK sample (preferably with own and parental EA & birthplace measured) are necessary in order to investigate the discrepancy in direction of effects between the UK and Dutch population, and the role of causal variants therein.

Non-random mating in human populations can be driven by heritable social traits like religion and EA through migration and assortment. The impact of these mating behaviors on the genomic structure of a population is not always directly captured by traditional measures for population stratification, such as ancestry-informative PCs. These findings are relevant for genetic association studies, since these behaviors can be associated with additional traits of interest, like psychiatric disorders with religiosity [1,48], or IQ with EA [22]. Deleterious effects of inbreeding studied by associating *F*_roh_ and the trait of interest usually require much larger sample sizes for detection than that of the current dataset (~12,000–65,000) [24]. We suspect that ancestral behavior may have influenced genetic variation more systematically than genetic variation influenced the current measurable behavior in our dataset. This additional confounding and non-causal “noise” may have contributed to the difficulty of finding consistent genetic association signals for many behavioral traits, especially if the nature, effect size, and/or direction of such confounding effects would differ per population. We recommend that cohorts contributing to meta-analyses of genetic association studies on behavioral, cognitive, and social traits search for patterns of variation caused by the social/historical context of their population, so these can be accounted for accordingly in their analyses and interpretations.
